# Navigating the discourse on the use of social robot Pepper: ethical and practical implications

**DOI:** 10.1186/s12877-026-07640-2

**Published:** 2026-05-14

**Authors:** Tenzin Wangmo, Sophie Sieber, Yi Jiao Angelina Tian, Delphine Roulet Schwab, Bernice S. Elger

**Affiliations:** 1 Institute for Biomedical Ethics, University of Basel, Bernoullistrasse 28, Basel, 4056 Switzerland; 2 La Source School of Nursing, HES-SO University of Applied Sciences and Arts Western Switzerland, Avenue Vinet 30. Lausanne 1004, Vaud, Switzerland; 3 Institute for Legal Medicine (CURML), University of Geneva, Rue Michel-Servet 1, Geneva 4, Geneva, 1211 Switzerland

**Keywords:** Robotic care, Technology acceptance, Tech developers, Policy makers, Qualitative study, Switzerland

## Abstract

**Background:**

Social robots such as Pepper are increasingly researched as potential tools that could support the care of older persons. Our goal is to highlight the multiplicity of perspectives and varied rationales held by participants in Switzerland for accepting or rejecting Pepper when used for caregiving purposes.

**Methods:**

Qualitative one-on-one interviews were completed with 116 persons living in two linguistic regions of Switzerland. The participants were either (a) older persons; (b) family caregivers; or (c) professional caregivers. Data related to the social robot, Pepper, from the interviews were sorted and analyzed thematically for this paper.

**Results:**

Themes highlight the different reasons for rejecting the use of Pepper, as well as finding space for it when necessary. Specifically, the rejection of Pepper stemmed and varied from just not wanting to use the technology, to perceiving oneself and older persons as being “too old”, to having ethical concerns and the omnipresent fear of the replacement of human care. Nevertheless, upon realizing that such social robots might *have* to be used, participants also discussed some of the advantages for certain groups, tasks where robots may be useful for, as well as the notion of robotic use as a second-best option.

**Conclusions:**

Not all older persons will accept the use of robotics tools for their care, even when they may be in need. This study discovered a generational rationale for refusing such robotic care. Individual autonomy to use such tools, costs, and their added value in addressing practical gaps in the lives of older person were important factors to consider when making policy decisions, as well as, in developing technology. There is therefore a greater need for policy makers and technology developers to consider the heterogeneity among the older adult population.

## Introduction

With the rapid development of robotic technologies, there is increasing attention to apply their capabilities to care and support older adults both in the community and in long-term institutional care settings. Autonomous robots that could address and adapt to the varied caregiving needs (e.g. social, emotional, physical, medical) of older persons are yet to be available [32]. There are, nevertheless, different robots have been in development and a few in use for specific purposes to support older persons [2, 33]. These include animal-like robots such as the therapeutic robot Paro, CuDDler, Golden Pup, AIBO, Robin, Miro designed for emotional support, social experiences, and reminiscing prior memories [9, 25, 33, 56, 60]. Paro, for example, is evaluated as having positive impact on older residents, particularly those living with dementia [37]. However, the use of such animal-like robots also raises concerns related to infantilization and stigmatization [57]. Empirical research also revealed misalignments between older persons and technology developers on their preferences towards such companion robots, which pointed to greater nuances on how technologies is framed as a solution for elder care and the importance of user-centric designs [6]. Furthermore, a recent systematic review on 23 randomized-controlled trials showed that such Pet-robot Interventions did not show clear and significant effects to reducing depressive symptoms in older adults aged 65 or above [54].

Humanoid-like robots, such as the Pepper, seek to engage, communicate and provide companionship to its end-users in a more personalized manner according to the needs and preferences of older persons [60]. Pepper was designed in 2014 by a Japanese firm. It is 1.2 m-tall and is equipped with voice features, emotion recognition, capabilities for expressive body language, as well as large wheels for mobility within controlled environments [35]. Pepper is thus designed with the capability to respond verbally and physically to human users, listen to expressions of emotion and repetitive topics, follow or accompany consumers around the home, navigate social platforms to allow telecommunication, provide reminders or schedule events, or present news or entertainment on demand [30, 44]. It includes a wider set of social capabilities than Paro, which provides a specific assistive function to soothe or entertain older persons [4, 24, 25].

To understand the uptake of robotic technologies, models such as the Almere are developed, which measure the acceptability of social robots in the care of older persons [18, 23]. The use of social (assistive) robots in the care of older persons have met with ambivalence, generating both positive and negative feedbacks related to its usability, maturity, aesthetics, role amongst existing alternative resources, as well as the overall attitude of robotics in care [19, 39, 40]. A scoping review by David et al., [11] indicated general positive feedback towards social robots to assist older adults, with particularly welcoming attitudes from older persons and lesser so from healthcare professionals. The latter worried about the replacement of human care in the already concerning trend of social isolation in older ages, as well as their own emphasis on the provision of care that should remain human. This worry that robots may replace human care has been repeatedly noted in several articles [12, 45, 52]. A more comprehensive review by Leineweber et al., [29] encompassed the ethical considerations for social robots in elder care across 248 articles, where they addressed issues such as the infringement of privacy, potential risks of oversight on certain stakeholders over others, perpetuations of social stereotypes from its humanoid shape, as well as the conceptualization of ‘care’ or ‘good care’. Other factors include its costs, doubts about its usefulness, concerns that it may harm the user, as well as autonomy and conflicts in decision-making to integrate robots in care [17, 22, 27, 39].

As robots most likely will (have to) be accepted in the future to support the care of older persons, many studies provide useful indications about different functions they could fulfil. For example, one study noted that robots can be a source of information [42]. Others revealed older adults’ preference of robotic assistance for more repetitive tasks and information management, and underscored the wish for human assistance when it comes to personal care [38, 47]. At the same time, a theoretical article argued that older persons may want robots for personal care since it preserves their social dignity [16], which was also reported by a few participants in an empirical study [15]. Studies with older persons teased out desired tasks for robots, which included practical activities such as holding people, lifting things, cleaning, grabbing, fetching objects, and mowing lawns, but wished for companion like activities to be carried out by humans [19, 52]. However, a Finnish survey elaborated on the views of care professionals, where respondents expressed skepticism and reluctance at the capabilities of robots to sufficiently carry, wash, toilet, and provide emotional relief to older persons [41]. Therefore, the robots as designed may not be able to fulfill the end-users’ care needs [30, 35, 53]. It also needs to be acknowledged that acceptance levels may differ between the age groups within the older population: The young-old of 65 to 74 years of age, the old-old of 75–84 years of age, and the very-old of 85 years and older [55].

In the case of Switzerland, there is a rising number of scholarship that underline the social and ethical acceptance of smart technologies. For instance, a qualitative study examined acceptability factors against technology acceptance models for different types of smart technologies among a sample of participants [15]. Two studies using the animal-robot Paro [37, 57] revealed that such robots are beneficial, particularly for those with dementia. An earlier study reported that among visitors of a robotic exhibition, there was overall positive image of robotics and they were preferred to contribute towards small tasks [1]. Additionally, a national study from Switzerland found that robots for assistive functions were more accepted than those for companionship, which concurs the studies presented above [58]. Finally, upon examining the description of humanness of care that robots are deemed to lack [31], the authors noted that there may be certain care circumstances when using robots may be advantageous to its older care recipient. To add to the limited knowledge that we have from the Swiss context and considering that our results could potentially be useful for similar contexts, this paper addresses the varied rationales for accepting and rejecting the robot, Pepper. Our goal is to highlight the multiplicity of perspectives held by Swiss participants when asked to potentially evaluate such a robot’s use in the care of older persons.

## Methods

### Ethics

The research study was evaluated by the Ethics Commission of Northwest and Central Switzerland (EKNZ) under ID: AO_2020-00027. All participants received an information document detailing the purpose, procedure, and anonymization measures before the interview date. After explaining the study details during the agreed-upon interview time and after receiving their written informed consent, the interview process began.

### Interview guide

The interview guide for the exploratory qualitative study included (a) general questions to understand the participants and their care needs (or care provision in the case of caregivers); (b) specific questions for each of the technology followed by (c) their general recommendations for the use of these technologies in the care of older persons. The technologies that we discussed during the interviews were emergency alarm bracelet, wearable sensors, ambient (visual) sensors, robots Pepper and Paro, and VR glasses. We brought pictures of these technologies during the interviews to ensure that participants were interpreting the technology as intended. In the case of the robot Pepper, we also showed the participants a video clip from YouTube^1^. Participants were subsequently asked a series of questions assessing their (a) opinion about this robot; (b) what they think about Pepper as a companion and rationales for and against such use; (c) their concerns and opportunities with using Pepper in the caregiving context; (d) and about problems and advantages of using a Pepper that is personalized to speak and react like, for example, a family member The interview guide originally written in English was translated to German and French by bilingual research assistants, finally back translated and checked to ensure content accuracy.

### Participant recruitment

In the German-speaking region of Switzerland, participants were recruited purposively using advertisements and contacts with several organizations caring for older persons, and snowball sampling. In the French-speaking part, participants were mainly recruited through personal and professional networks of the research team members. Eligible study participants were (a) Older persons over 65 years of age receiving care at home or a nursing home or an assisted living facility; (b) Family carers providing support to an older person, either a parent, grandparent or a spouse; and (c) Professionals working in nursing homes, home health care agency, and assisted living facility.

### Data collection

Three native Swiss interviewers trained in qualitative methods and interviewing skills conducted all the interviews for this project. One interviewer (NF) was completing her PhD at the time, the second (VD), was finishing her master’s education in medicine, and third (SS) was a trained nurse researcher. They completed the interviews in participant’s homes or a place of their choosing or via online videoconference. A total of 116 participants were recruited (Table 1 provides demographic information by linguistic regions). These interviews were between 46 and 189 min long. Study participants chose the venue for the interviews (mostly their homes). The interviews took place between September 2021 and June 2023. Professional caregivers included medical specialists, registered nurses, nurse specialists, care management employees, social workers, dementia care workers, and homecare specialist coaches. Some of whom care for older persons in their own homes, and other in home care facilities. Informal caregivers vary between those caring for older persons with dementia, and those without.

**Table 1:** Participants characteristic (*N* = 116)

German-speaking region (*N* = 67)	Older persons (*N* = 27)	Professional caregivers (*N* = 23)	Informal caregivers (*N* = 17)
*Age*	87.4 years (73–97)	45.2 years (31–63)	57.1 years (26–83)
*Gender (Female: Male)*	15 : 12	19 : 4	13 : 4
*Living / Working /* *Care situation*	Home: 13	Home care services: 9	General caregiving: 8
	Nursing homes: 10	Nursing homes: 9	Caregiving for someone with dementia: 7
	Assisted living facility: 4	Other: 5	Care recipient in nursing home: 2

French-speaking region (*N* = 49)	Older persons (*N* = 18)	Professional caregivers (*n* = 17)	Informal caregivers (*N* = 14)
*Mean Age (range)*	87.4 years (78–95)	42.7 years (29–62)	64.0 years (53–84)
*Gender (Female: Male)*	13 : 5	15 : 2	8 : 6
*Living / Working /* *Care situation*	Living at home: 8	Home care services: 4	General caregiving: 14
	Nursing homes: 2	Working in nursing homes: 4	
	Assisted living facility: 8	Other: 9	

### Data analysis

Each interview was transcribed verbatim by the same interviewers. For this paper, all coded segments corresponding to the robot Pepper were extracted in MAXQDA. The first author read and coded the entire Pepper dataset and examined all the codes within the topic of this paper, acceptance (or lack thereof) of Pepper robot. This led to an iterative process of re-coding the data to build themes and sub-themes that explain the range from rejection to possible acceptance of this robot. The data analysis followed the procedure penned in the thematic analysis [7] supplemented with reflexive thematic analysis [8]. The preliminary findings were presented to the co-authors, who provided edits and explanations to either concur or challenge the preliminary findings. The analysis presented below includes quotes from the participants, with shorter quotes embedded with the paragraph (within quotation marks) and longer ones are presented after the paragraph. They are translated into English and edited to ensure readability. We use DE and FR to indicate the language region, that is German-speaking and French-speaking, respectively.

## Results

In the analysis of the data related to Pepper, several nuances were illuminated. These were associated with whether Pepper is deemed appropriate for the intended older adult population with care needs, what it means to use a robot care provider, and discussions surroundings “better” ways to integrate robotic tools in the caregiving space for older persons. There were little differences in the themes and sub-themes (Table 2) discussed by participants hailing from the two linguistic regions. Also, most of the issues were raised by members of the three participant groups, albeit some raising one issue more often than others. However, these frequencies were not of interest for our qualitative analysis.

**Table 2:** Using robot Pepper to care for older persons?

THEMES	1. Reasoning rejection	2. Warming up to robotic care?
SUB-THEMES	Clear rejection of Pepper	Pepper for specific groups
	Too old for Pepper	Pepper for specific tasks
	Pepper, not for the moment	Second best option
	Ethically relevant worries	
	Replacement of human care	

### Reasoning rejection: from individual attitudes to value judgements

#### Clear rejection of Pepper

Among the study participants from both linguistic regions, a few, mainly older participants, can be categorized as those who felt that they must reject the use of Pepper. One older participant refused to even watch the YouTube clip of Pepper. Another professional caregiver participant stated that robots in general are “terrible, I think that’s terrible” (DE_Professional Caregiver31). Such responses imparted a strong emotional feeling of the robot being undesirable for their current living situation, along with a wish that they will not have to use it. Along this same line of reasoning, personalizing Pepper to mimic the voice of a loved one was also generally rejected. However, they were not against others using the robot, should others find it useful.

...playing with something so stupid all day. … There are ones that look like a person, but I don’t want that either. No, I’m far away from that, far, far away. That would be the last thing - I hope I don’t live that long, that it will be introduced in the old people’s home. (DE_OlderPerson21_nursing home)

#### Too old for Pepper

Many participants from the three population groups in both language regions underlined that Pepper will be rejected or cannot be used for the very old persons [presumably those who are 80 years and older; the average age of the older participants was 87 years] as they were “too old”. Here, what the participants revealed was a generational issue, where due to their life experiences and socialization, it will be challenging for very old persons to accept such a robot. For instance, an older participant living in nursing home (DE_OlderPerson61) emphasizing his old age, said, “And I think so - I’m just old” and another (FR_OlderPerson121_nursing home) stated, “Because I don’t like talking to a robot in the abstract, I’m not into robots”. A family caregiver (FR_InformalCaregiver141) reported that the mother would reject Pepper by simply not using it, “She’d put it in a corner, I think,” and along the same way, another family caregiver providing general assistance (DE_InformalCaregiver81) noted, “So I don’t think that Pepper would be used much…. She’s not someone who…. She likes to read a lot and she thinks that’s good. But that’s it.” Underlining the unfitness of Pepper for the (very) old generation, a professional caregiver serving older adults in their homes (FR_ProfessionalCaregiver161) pointed that, “I don’t see any advantages. [laughs] And then no disadvantages. Well, I don’t see her [older person] using it, actually”. Within this “too old” argument was the underlying belief that it might be difficult to learn how to use new technologies and it might be an intrusion into the lives of these very old individuals.

It’s too intrusive. What’s new for these people is that explaining something simple to them, explaining the iPad to someone who’s 80 years old, isn’t that simple. … But for us, it seems so simple. Even for me, who wasn’t born with it, the iPad is simple. So you can imagine, you put something like that with [a person who’s 80 years today]? No. (FR_InformalCaregiver31)

In a different way, but also relaying that robots are tools that participants are not used to, an older participant living at home (DE_OlderPerson151) said, “That is foreign to me”, while another professed that he likes simple things and does not wish to “be bogged down with so much stuff” (FR_OlderPerson_31). Similarly, putting himself in the place of a very old person, a family caregiver (FR_InformalCaregiver131) stated that he would also reject Pepper, “Well, if I were 80 years old today, well, no, really, I don’t think so.” Within the above discourse on the generational issue, the participants somewhat relayed an inappropriateness to use Pepper for this group of very old persons. Several of the participants rejecting Pepper for generational reasons also felt that the next generation will be more open to accepting it because they have become familiar with technologies.

No, I find that a bit surprising. It disturbs me a bit, because especially now, maybe for older people, like us when we’re 80 or 90, in fifty years or so, well people, young people will already have been more in contact with technology and so on, so maybe it will seem completely normal to us, but older people today, I find that there’s a really huge barrier with technology, and even more so with robots. (FR_ProfessionalCaregiver171)

#### Pepper, not for the moment

 Unlike the above rationales for rejecting the use of Pepper, another sub-theme evident during the analysis, was a temporary rejection. By this, we mean that, at present, participants do not have a need to use a robot. They reported being in a physically and mentally good health and hence no support was required in their current situation. Also, they wished to remain independent and do activities on their own: “I still like to go somewhere on my own initiative” (DE_OlderPerson311). Likewise, a family caregiver (FR_InformalCaregiver31) felt that the older person will not use Pepper as long as she is somewhat independent and the use of a robot was seen as a stage of high dependency where she would need to go to a nursing home: “Not as long as she’s mobile. … Then again, if she can’t stay for a while when she’s too…, when she absolutely needs the robot, then she goes on to the next stage [nursing home].”

Responses were similar when participants were asked about their attitudes if they received a Pepper as a gift. Those who felt that they were healthy and not in need of technology would give it away and perceived such a gift as waste of money. One older participant (DE_OlderPersion41) pointedly replied, “I would give it to someone who could use it. I would bring it to someone who has dementia”. There was also openness to use Pepper when need for support becomes a reality, (FR_OlderPerson611) “if I had a limitation that meant the robot could do things for me, help me, then fine, but not like… that’s why the companion type wouldn’t go down well with me”. (This notion of using robots for certain activities and not as a companion is discussed later.) Highlighting the temporal rejection, another participant recounted his or her general rejection of technology when initially introduced, but gradual acceptance when needs became more evident.

But you know, I said no to everything in the beginning with additional aids and now I have a stairlift, which is also a robot, I have a bed that can be adjusted up and down and all sorts of things, it’s also a robot. … And I always said no at first and then afterwards I thought I would have preferred to have said yes much earlier. (DE_OlderPerson_612)

#### Ethically relevant worries

Related to this theme, several risks to the user were specified that may hinder the use of robotic technologies. These include the risk when an older person (a) self-isolates with robots, (b) over relies on technology, or (c) loses control over technology. Concerning risks of isolation with Pepper at home as well as in nursing homes, the worry was that when it becomes difficult to find the company of other humans, people may find it in a robot. Concerning the tasks that Pepper can perform to support older persons, the participants worried about becoming over-reliant on Pepper’s support. They felt that such situations decrease their abilities to perform tasks for themselves and thus increase their reliance on an external tool. A unique concern, which came up only a few times, was the fear that the technology may go out of control and harm the user. For instance, a family caregiver (FR_InformalCaregiver131) said, “Well, if all of a sudden the… the robot goes off the rails and then decides to strangle you.”

Well, I don’t know, I can’t tell you at first sight no, but… there are lots of… of… technical means where it generates a kind of dependency, for example, I… I don’t watch TV because I don’t have the time, but I know that there was a time in my life when I had TV… “Oh yeah, come on, let’s watch the news. Oh yeah, I’m going to look again to see if” there’s an extraordinary potential for [it to be] a source of addiction so maybe I could become addicted to Pepper, not addicted to Pepper, I don’t know. I find the main thing sad like that. (FR_InformalCaregiver_121)

Another general worry was the cost associated with buying such a technology revealed by some participants. An older participant (FR_OlderPerson_131) said that her daughter would not have additional resources to buy Pepper for her. Similarly, another participant noted, (FR_ProfessionalCaregiver_121) “Well, of course there’s the financial side, and I think that’s important for a lot of our patients too,” with others raised the problem of who would finance such an expensive technology.

Finally, another issue that was discussed but only a few times and by caregiver participants from the French-speaking part was data protection. For instance, a professional caregiver working in nursing home said, (FR_ProfessionalCaregiver71) “ … at the same time he’s a robot, he only knows what we give him, so it’s up to us, once again, to set… to set the limits, and what limits that is”. Along the same lines, another participant thought about potential ability of Pepper to record discussions, which meant that there will be privacy concerns.

#### Fear of replacement of human care

Almost all participants from both language regions reasoned that social robots like Pepper lack humanness and cannot be used in the caregiving space, which is an inherently human and interpersonal space based on relationships. Attached to this line of reasoning was the fear of replacement of human care. Participants viewed this replacement as a negative outcome for older persons, who, they felt required support from family and professional caregivers. For example, a family caregiver stated, (FR_InformalCaregiver_131) “Yes, yes, yes, it’s really great. No, but it’s… it’s great technology, but if it has to replace someone close to you. Yeah, I think it’s a bit sad.”

Talking about the specific issue of Pepper lacking humanness, participants were quick to note that it is an object. Pepper is not natural, because it is neither a person, nor can talk like humans and is not intelligent: (FR_OlderPerson411) “Yes, that’s it, it’s not a human being, that’s it exactly, absolutely”; (DE_OlderPerson61) “So the fact that you’re moving away from the natural, …No, you can’t replace that. So it should actually be logical. They are just artificial figures…”. Without such human elements, a robot was seen as an object that is not able to form an emotional bond. Furthermore, a robot’s touch and sound appears cold, whereas that of a human is warm: (DE_InformalCaregiver11 caring for older person with dementia) “That when you touch it, it’s cold…. Yes…. It might be fun for people to see how it works, how it moves. But I don’t see it as an interpersonal relationship at all”.) Repeatedly brought forward was also the rationale that humans are extremely social beings and thus would not function well with lifeless robots. A few relayed that they would not talk to objects that cannot think because generally they do not talk to other objects in the house, like a furniture: (FR_OlderPerson411) “For me it’s a piece of furniture, that’s exactly what it is. There’s no human contact, you can’t babble, that’s it.”

As aforementioned, replacement of human care by robots was a concern. Many participants stated that older persons need human care because, (1) care is an interpersonal activity, and (2) humans are social beings, and (3) the older generation is used to and expects this type of care. They highlighted their fears towards an erosion of human care. For example, an older participant living in nursing home stated (DE_OlderPerson101) “No. So I don’t want to be woken up by the robot in the morning.” And a family caregiver of a person with dementia noted (DE_InformalCaregiver_71) “And if you bring one of these to an afternoon, a games afternoon, then that’s fun, I think. But as a daily use, I think it’s a shame. Because I just don’t think it should replace the human touch.” Realizing that Pepper-like robots could be interesting options from an economic perspective, participants still believed that it was uninteresting from a human perspective. Along the same lines, family caregiver stated that they would not welcome news that nurses caring for an older parent in nursing homes have lost their jobs and replaced by robots, (FR_InformalCaregiver51) “I’d still be shocked to think that the nurse my mother knew, etc., lost her job last week. … from now on it’s Pepper who… who comes every morning …, I’d be upset.”

Overall, study participants agreed that human connection must continue to be encouraged and replacement with technology resisted, as there was no substitute for human contact. Moreover, when asked participants if they would use a robot Pepper should they receive it as a gift, a professional caregiver said, (FR_ ProfessionalCaregiver_151) “… honestly, I’d rather see real people than a robot. I’d rather say to my family, “Instead of giving me a robot, come more often”. I think that would be more my reaction.” In the response of this participant, there was a concern that using Pepper might become a leeway for children to do the easy thing, that is, give a robot instead of taking efforts to visit regularly. Another participant warned that Pepper could be used by professional caregivers without thinking twice to make their work easier.

If you want to make it easy for yourself. So, if a nurse thinks, oh come on, instead of bringing this person the tea myself, I’ll just send it [Pepper] and then I have less to do and can, I don’t know, sit on my cell phone or something, then it would be wrong, I think. (DE_ InformalCaregiver311 of person with dementia)

### Warming up to robotic technology?

#### For specific groups

In addition to activities that Pepper can fulfill for older persons and their caregivers, study participants mainly discussed the benefits that a specific group of older persons who are alone may derive from it. A family caregiver stated that, there was no need for Pepper for his own parents, both of whom are still alive and are living at home. Additionally, older adults living in nursing homes were viewed as being “lonely”, whose families are not able to visit them often, and with shortage of staff, they gain limited social contacts. In such situations, a Pepper that is available to talk and show some interest would be valuable. An older participant living in nursing home (DE_OlderPerson91) described “… a lot of people feel lonely and until they get used to the fact that we’re all here [in nursing home] together, …, that a robot like this might be helpful for conversation.” Within the discussion of using Pepper in the nursing home setting, participant raised its entertainment value as an object of curiosity. Pepper could thus bring together a group dynamic where residents may be more willing to engage in something different and new, which may have a better outcome for the residents.

It’s the loneliness, of course, that the old people have, and I realize that every day, that they’re lonely when the family doesn’t have time to come every time, and that’s replaced with a robot. But at least you can see the face of this lady who…yes, that’s the loneliness they have and when a robot like that talks to them and gives them attention… is valuable to them. (DE_ProfessionalCaregiver41)

#### For specific tasks

Our analysis indicates that there are pockets of activities where the use of robots may enjoy more acceptance than using them as companions. A caregiver participant generally rejecting Pepper felt that it could be used for **household activities**, (FR_ProfessionalCaregiver151) “No, or unless they manage to get him to help with household chores”. In a similar vein, many participants reported that Pepper can be accepted to perform specific tasks which were mostly practical in nature comprising activities such as cleaning, picking things up, bringing tea, water, coffee, cooking meals, making bed, support with wearing shoes, cleaning the house and giving reminders. Keeping or putting Pepper at that level of “**service provider**” appeared as the “safe” space of robotic use in the care of older persons. As an example from many recurring practical tasks, an older participant living at home (DE_OlderPerson132) relaying the use of Pepper stated, “So if I were at home now, for example, I’m bedridden, I can’t get up at all, that I can say to him [Pepper], “You, bring me a glass of water. Or bring me my tablet”.” Another older participant living in nursing home (DE_OlderPerson411) felt that some ability of the robot to notice an older person’s lacking capacity to do something and thus reacts to the situation would be desirable, “You see, he’s getting up now, he can’t do it himself, then the little one [Pepper] can say, “Did you forget the walker?” or “Take the stick with you” or things like that.” As it is nevertheless clear to the participants that robotic technologies cannot perform such tasks at the moment, an older participant noted the need for continued technical developments, (DE_OlderPerson111) said, “take butter out of the fridge - simple example - or take the jam out and unscrew the lid and spread the bread with butter”.

The use of Pepper for the specific tasks was also viewed positively if it could **support professional caregivers** in different ways that would provide reprieve to the caregiver as well as make them more content with their responsibilities. This included, for example, accompanying an older person to use the restroom. A family caregiver (DE_InformalCaregiver_101) reporting this possibility relayed, “if you could tell Pepper to accompany someone to the toilet and support them and hold them and do it, … then I think why not? I mean, we usually do that in everyday life too.” An older participant (DE_OlderPerson141) revealed the potential of Pepper to address tasks that the caregiver would rather not do: “Of course, I’d rather have someone who could do things in care, for example, who could change your diapers or something, which is unpleasant for the person who has to do it, for example”. Emphasizing the care stress could be reduced, a participant (FR_InformalCaregiver_81) put this as such: “Well, that means she wouldn’t feel so lonely at home, for one thing. And that way, I don’t have to go and see her all the time. Because sometimes, it’s true, I get a bit stressed.”

In the context of care? …, if you like, that a guy could use to carry heavy loads. I think it’s great because, physically, you can… you can save yourself some effort. For you nurses [referring to the interviewer], for example, we know that turning people who are very heavy or very ill is not good for your back. In the long run, you’re the one who has problems. I think it can help, especially now that there’s a fairly glaring shortage of staff. (FR_InformalCaregiver_711)

#### Second-best option

Highlighting ambivalence when it comes to discussions surrounding the use of Pepper, a theme that embeds within the specific tasks robots can do as well as fear of replacement of robotic care, was this idea of “having to” accept Pepper-like technology. We found this theme repeatedly in the data from the German-speaking participants. They discussed it as “no other option” in light of limited care staff and continuing technological developments, which may in some ways normalizes robots application.

And that will happen! So, I’m convinced of that, and I don’t even think that will take so long … because there’s so few staff, that’s a whole reason. I can certainly imagine that - or brings food, that the staff don’t have to come. That that it is programmed to come to the woman (name), room so and so, ok. I am convinced that this is coming. (DE_ OlderPerson101)

Along this vein, participants said that the role of human appears to be vanishing with technologies developed to take over tasks that only humans performed (e.g. the service/servant tasks). Many participants also questioned whether there are enough humans available to care, both now and in the future. Moving along these concerns led to the conclusion that when there are no or very few persons available to provide care for older persons, then it makes sense to replace their roles with robots. An older participant (DE_OlderPerson81) coming to a realization stated, “… I’m saying maybe that could be quite good (using Pepper), it’s possible, but I don’t want that now. Of course, if there’s nothing else but something like that, you have to accept it, I suppose.” Here it is important to underline that the replacement was a second-best option. More specifically, when there is the option, it would be much better to have human-to-human care.

I mean…. perhaps people are already getting a bit lost. But the question is, are they there or not? If there are no people in elder care anyway, or too few people, then I think it makes perfect sense to replace them. But now…. I mean, if you have other options, then I think it’s certainly nicer if you have person-to-person care, if that’s there. For sure. But I can well imagine using it as an add-on. But I think it would be a shame if it went completely without other people afterwards. (DE_InformalCaregiver101)

Nevertheless, participants stated repeatedly that the ideal situation is where human contact is not replaced and robotic care is used where needed as an add-on. Such an option may make caregivers work qualitatively better, which is then a win-win situation. The preferred situation would then be where robots are able “to free up time, so that people can have… I think that the older we get, the more we need human warmth, to be able to talk” (FR_InformalCaregiver31).

Personally, I don’t see it as a substitute, but ultimately, it’s something additional, it’s another tool. … The robot is actually additional and can, for example, cover the things that care [workers] can’t do, or in some cases simply… For example, the robot doll for stroking, I can’t expect a carer to stroke a little bit to that extent, that’s not possible. And there it’s like something extra. (DE_ProfessionalCaregiver311 working in nursing home)

Moreover, in a context of a nursing home, where one underlying presumption was that older residents do not receive enough attention, the use of Pepper was deemed to be a welcome one. In an ideal situation, Pepper is able to fill-in for the professional caregiver when needed and act as an add-on support tool. As an example, a family caregiver participant (DE_ InformalCaregiver_41) suggested: “so if the carer has to go to the toilet and has to leave the group alone, which is often necessary, then it’s a good idea to entertain them a little [with Pepper].” Such targeted use will also reduce the burden of care for those professional care personnel while Pepper takes up other practical tasks, and also “does not become a substitute for a person” (DE_InformalCaregiver71). Concerned about the lack of staff in nursing home, an older participant (DE_OlderPerson_101) opined, “Yes, well, he can bring them the food so that not so many staff have to do it themselves. Why not? Or the medication, why not give it to him and say, “Go to Mrs. (name) now.” At the same time, a family caregiver participant felt that Pepper could be useful for care providers in a way that it addresses the “difficult patients”. Understanding that Pepper is a new technology in the nursing care context, a participant underlined that there has to be rules and expectations clear to everyone so that the benefits can be maximized.

I don’t know how it could be used. Maybe use it for very specific activities, for animation or something, that could be interesting, but where there’s always the animator there and then the robot, but not just the robot in fact, it’s… And then I think it could be fun, it could be nice to do an activity with it. But it shouldn’t replace the person. (FR_ProfessionalCaregiver_21)

## Discussion

The findings add important information to the limited knowledge about social robots like Pepper in the context of Switzerland. Our extensive findings on one type of robot, Pepper, enables nuanced understanding on acceptance or lack thereof of this robotic technology and substantiates previous literature on where such social robots may find greater levels of acceptance when used in the care of older persons. Our Switzerland-specific context thus adds to the geographic breath. We highlight results that cautions against when and how such robotic tools could be dispersed in the care context to be most beneficial.

Firstly, we are able to substantiate the findings related to ethical issues, with respect to the choice of using (or not) robots within the care situation the older person finds themselves. Although some participants may not currently want the robot for themselves, they are not against others using it. As a way to reject the use of robot in its current form, participants delayed their potential interest to a later date, or to an older version of themselves that may require the functionalities available in a robot such as Pepper. This is not a new finding and has been echoed in previous studies [38, 53]. Thus, the choice to use or not use a robot [49] remains an important factor in technology uptake in the community setting. However, such choices may be limited in institutional settings where robots become part and parcel of care support. From the literature, we know that such choice to institutionalize robotic care can be, for instance, due to perceived benefits particularly for those with dementia [37, 57]. An older person with dementia might “like” and benefit from Pepper while someone without dementia could feel humiliated and refuse the use of such robots [21]. At the same time, we suspect that there may be acceptance in cases where older person wishes to avoid humiliating and socially painful situations when very personal care is provided by a caregiver [16]. There is a tendency to designate another population group, such as those persons with dementia or those who are lonely, as more suitable for a technology, thereby redirecting interest rather than for themselves as end-users.

Secondly, cost issues related to who can afford a robot were also mentioned, but remain unaddressed also beyond Switzerland, as these robots are neither readily available nor are easily affordable tools [14, 39]. The high cost also means that their use is currently limited to the few who may afford them, either personally or in contexts where public policy covers for partial or all costs [52, 59]. Nevertheless, if robotic technology becomes affordable in the future and could be replacing human care because it becomes a financially better option, we have to address the risk that care is entirely provided by robots. From an ethical point of view, such a situation is said to be undesirable [50, 51].

Thirdly, robotic technologies due to their lack of saturation in the Swiss market (and elsewhere), are likely to face some levels of rejection in light of the diversity that is evident within the older adult population. Based on our interviews, we attributed the rejection visible among the study participants (average age 87) to *generational aspects of rejection.* This rationale is an important one to consider before implementing policies to accelerate robotic care tasks at institutional and national levels. It is different from rejections due to a dislike of technology, ethical, social or technology-specific reasons, and lack of current health needs necessitating such support, which have been reported elsewhere [17, 20]. We know as well that for any technology uptake, there is a minority (less than 20%) who refuse or are late to accept technologies compared to the rest of the population [43]. It is known that the rate of acceptance also depends on issues such as exposure to new innovations and familiarity with similar technologies. In our study, many participants spoke about being “too old” to learn the technology and “not being used to it”. Our conclusions do not, however, suggest that very old persons *cannot* learn to use and become comfortable with new technologies. We also point to differences among those who are older, additionally varied by characteristics such as age, education, and affluence, which impacts technology use [48]. Technology use will also depend on their contextual factors of a specific country, experience using the technology [26], as well as individual personality and goals. With the generational rationale, we want to emphasize that there are values and expectations that different generations imbibe, as part of their socialization and aging process, which are important considerations in technology dispersion. One could argue that this generation of very old persons were socialized at a time, when their expectation for their own old age was human care provided solely by a human caregiver. This may explain the reason that participants insisted on human care and talked at length against the replacement of such care, a sentiment that has been widely cited [52]. Fulfilling this value may be more important for this generation of very old adults, whereas the care provided using robotic technologies may receive greater acceptance with the subsequent baby boomer generation [5]. This generational reasoning for rejecting robotic technologies leads us to suggest (1) realistic assessments of the specific availability and target audience of a technology, and (2) technology development and its future use must also be cognizant of variations among older adults due to their values, socialization, health characteristics, technology competences, and other factors that are important for technology uptake. However, it is critical not to adopt an ageist stance by lumping all people aged 80 and over together and assuming that they are inherently resistant to robots and incapable of using them.

Fourthly, as evident from the discussion above, participants were worried about the potential for the replacement of human care by robotic technologies. They are nevertheless aware and recognized that, in light of the growing older adult population, it would be necessary to balance certain wishes and realities when it comes to technology rejection. What remains clear from the study results is the notion of “humanness” that must not be replaced (see [31]. Thus, as part of their second-best option and supplementing human care, the study participants seemed content to let the robots carry out practical care tasks that may be boring for humans, or those chores that are usually devoid of social or personal interactions, e.g. cooking, laundry, bringing food, cleaning etc. These points were also found in previous literature [19, 52, 53]. Participants were less positive when Pepper ventures into the role of a friend or a mate [3, 10]. With increasing development in the field of artificial intelligence, it is likely that robots may have more capacities than what we can imagine today. From our findings as well as that of others, it is prudent that technology developers find ways for such robots to also perform some practical tasks, which could enable the user to find additional reasons for acceptance in addition to having a conversational mate. This could include practical things mentioned above, as well as developing robots to provide some physical touch, which may have physiological benefits (calming effect) for the end-user [13].

### Limitations

Our study has several limitations. Similar to other studies that assessed *a priori acceptability*, our participants were only shown a picture of Pepper and a video clip of Pepper rather than having an in-person experience with the robot [11, 34]. Many of the participants were also unfamiliar with Pepper prior to the interview. While we believe it pertinent to acknowledge this as a limitation, we do not however deem this surprising. Robots like Pepper are expensive tools and remain often unfunded by insurance companies, are thus not yet widely available nor saturated in the market [14]. One cannot simply venture to a store and purchase Pepper, unlike a smart wearable watch or other monitoring devices. However, because of this focus on *a priori acceptability*, we also acknowledge that existing literature has indicated a potential improvement in the views of social robots after increasing engagement [28]. Secondly, despite the large sample size (116 participants), it is still a group that was recruited purposively, some may have been affected by social desirability in stating what is deemed acceptable of robots, and thus we do not claim generalizability of our findings. Although we took care to include older persons with different profiles (age, gender, place of residence, level of education, etc.), the older adults who participated in the study were generally well integrated socially, spoke the language of their place of residence, and did not live in significant precariousness. It is therefore possible that more marginalized older adults (e.g., those from visible cultural minorities) may have different perceptions regarding the use of a robot such as Pepper in the care of older persons. Additionally, they were on average 87 years of age, representing the “very old” among the older population. Due to this sampling, we see the generational rationale of rejection. We have taken caution in ensuring that we interpret our findings carefully and not homogenize the very heterogenous older adult population.

### Conclusions and future directions

Our findings, first and foremost, underscore the importance of ensuring that older adults are not treated as a homogenous group on whom different technological tools deemed useful are imposed but as individuals whose needs, wishes, values, and current situations are considered carefully when technologies are used in their care. Also, we underline the fact that there will be subgroups of older persons who may not be the most suitable candidate for technology adoption due to many reasons. One such reason that we uncovered in our sample of participants is related to the generational aspects among our participant group. Many more reasons may however be found when greater diversity among participants is achieved. Furthermore, the development of robotic solutions should address actual care needs (practical support, physiological touch) of the end-user, rather than merely be a socially intelligent tool.

An issue that remained mainly unraised by our participants and was not directly addressed by our interview guide is the moral duty of the family caregivers to provide the expected care. Such duties could be imagined as some form of direct caring for the older person until the moment of passing, and where the use of robot is then viewed as a form of disrespect of this duty to care, independent of the autonomous choices of the older person. Whether it is morally wrong if older persons were “abandoned” to the care of the robots and what its consequences for society at large can be were not discussed with and by the participants. There are ethical discussions on this topic [46, 50, 51]. Nevertheless, it becomes necessary to critically evaluate the consequences of such robotic care on society. That is, if we accept the care of older adults by robots, would we then become a less caring society? Would we be normalizing the social abandonment of older adults or older family members? In case of a complete replacement of care by robots, there is risk that those who can afford human care may be those who are already privileged in the society in terms of socioeconomic status and resources. Are such new forms of hierarchy desirable? In addition, as it is often women who provides care work [36], would a shift towards robotic care be beneficial or harmful for them? These questions lead us to discussions surrounding the definition and significance of a moral society that we want to live in, which was unfortunately beyond the scope of this study. However, given the findings of this study and others, compounded with the realities of technological dispersions that we are experiencing, these become morally and ethically relevant questions to examine at the level of the family and the society.

## Data Availability

The interview guides and anonymized interview transcripts supporting the conclusions of this article are available in the SwissUBase repository, via (10.48573/gha2-k317) Dataset ref. 2778. Only the interviews where participants consented to sharing them on an Open Access Platform are found there. The interview guide has not been published alongside any other publications from this study and is only submitted to SwissUBase under the link above.

## Footnotes

1
https://www.youtube.com/watch?v=UMWkSQz3aOo&t=3s, shown until 1 min14s.
